# Eco-Friendly Synthesized PVA/Chitosan/Oxalic Acid Nanocomposite Hydrogels Embedding Silver Nanoparticles as Antibacterial Materials

**DOI:** 10.3390/gels8050268

**Published:** 2022-04-25

**Authors:** Irina Popescu, Marieta Constantin, Irina M. Pelin, Dana M. Suflet, Daniela L. Ichim, Oana M. Daraba, Gheorghe Fundueanu

**Affiliations:** 1 “Petru Poni” Institute of Macromolecular Chemistry, Grigore Ghica Voda Alley 41A, 700487 Iasi, Romania; impelin@icmpp.ro (I.M.P.); dsuflet@icmpp.ro (D.M.S.); ghefun@icmpp.ro (G.F.); 2 Faculty of Medical Dentistry, Apollonia University of Iasi, 700511 Iasi, Romania; danielaluminitaichim@yahoo.com (D.L.I.); oana_daraba@yahoo.com (O.M.D.)

**Keywords:** silver nanoparticles, PVA/chitosan hydrogels, oxalic acid, eco-friendly, antibacterial

## Abstract

PVA/chitosan (PVA/CS) composite hydrogels incorporating silver nanoparticles (AgNPs) were prepared by double-cross-linked procedures: freeze–thawing and electrostatic interactions. Oxalic acid (OA) was used both for solubilization and ionic cross-linking of CS. AgNPs covered by CS (CS-AgNPs) with an average diameter of 9 nm and 18% silver were obtained in the presence of CS, acting as reducing agent and particle stabilizer. The increase of the number of freeze–thaw cycles, as well as of the PVA:CS and OA:CS ratios, resulted in an increase of the gel fraction and elastic modulus. Practically, the elastic modulus of the hydrogels increased from 3.5 kPa in the absence of OA to 11.6 kPa at a 1:1 OA:CS weight ratio, proving that OA was involved in physical cross-linking. The physicochemical properties were not altered by the addition of CS-AgNPs in low concentration; however, concentrations higher than 3% resulted in low gel fraction and elastic modulus. The amount of silver released from the composite hydrogels is very low (<0.4%), showing that AgNPs were well trapped within the polymeric matrix. The composite hydrogels displayed antimicrobial activity against *S. aureus*, *K. pneumoniae* or *P. gingivalis.* The low cytotoxicity and the antibacterial efficacy of hydrogels recommend them for wound and periodontitis treatment.

## 1. Introduction

Silver nanoparticles (AgNPs) have attracted significant interest due to their broad antimicrobial, antifungal, and antiviral properties [1,2]. Their entrapment into hydrogels or polymeric membranes leads to the obtaining of antimicrobial composite materials with biomedical applications in wound and burn treatment [3,4,5], or periodontal regeneration [6,7]. The balance between the antibacterial efficacy and the toxicity of AgNPs is under debate; however, when AgNPs are entrapped into polymeric networks, their toxicity is reduced [8,9]. Different approaches can be used to obtain composite hydrogels containing metal nanoparticles: (i) ex situ approaches, including mixing the preformed AgNPs with the polymer solution before the cross-linking [5,10] or using functionalized AgNPs as cross-linking agents [11], and (ii) in situ approaches, including loading the Ag^+^ ions into preformed hydrogels followed by the reduction of Ag^+^ ions [4,12,13,14], or synthesizing metal nanoparticles during the cross-linking [15,16]. Hydrogel composites containing AgNPs for biomedical applications usually contain biopolymers such as chitosan [4,15], alginate [10], or carboxymethyl cellulose [9], due to their biocompatibility and wound-healing properties. Chitosan (CS), the only naturally occurring cationic polysaccharide, obtained by deacetylation of chitin, is known for its intrinsic antibacterial activity [17] which could provide a synergistic antibacterial effect in combination with AgNPs [18]. In addition, CS has the ability to reduce Ag^+^ ions to Ag^0^ and also to stabilize the AgNPs [19,20,21]. The obtaining of AgNPs in aqueous medium without the toxic reducing agent but in the presence of biocompatible natural compounds, including CS, is in accordance with the principles of green chemistry.

Hydrogels, 3D polymeric networks insoluble in water but able to absorb large quantities of water or biological fluids, are obtained by chemical or physical cross-linking of natural or synthetic polymers. They can closely simulate natural living tissues, having numerous biomedical applications [22,23]. Physically cross-linked hydrogels gained increased attention in the last decades in order to avoid the use of traditional chemical crosslinking agents. One method for the production of physically cross-linked hydrogels is the freeze–thaw processing, a method used for the first time by Peppas to obtain poly (vinyl alcohol) (PVA) hydrogels [24]. PVA is a semicrystalline synthetic polymer with good biocompatibility and low toxicity, known for its applications in contact lenses, cartilage replacement, wound dressings, or drug delivery systems [25,26,27,28]. The limited applications of pure PVA hydrogels can be enhanced by blending PVA with natural or synthetic polymers [25,26]. CS, widely used in wound treatment [29,30], can be blended with PVA to obtain wound dressings with good mechanical properties [25,31].

PVA/CS hydrogels obtained by freeze–thawing procedure were intensively studied in the literature [31,32,33,34]. Generally, the increase of CS amount in the composite leads to less cross-linked hydrogels with higher dissolution in water, higher swelling, decreased tensile strength, and Young modulus [31,33]. In order to increase the mechanical properties of CS/PVA hydrogels obtained by freeze–thawing method, an additional cross-linking of CS or of both polymers was performed, usually by gamma irradiation [35] or by ionic cross-linking of CS with SO_4_^2−^ anions [36,37]. Dicarboxylic acids are frequently used for the CS cross-linking through non-covalent interactions [38,39,40]. That is why our strategy involved the use of oxalic acid (OA) as supplementary cross-linker in order to enhance the stability of PVA/CS hydrogels and to immobilize silver nanoparticles enveloped in CS.

The addition of Ag or ZnO nanoparticles in PVA/CS hydrogels is generally used to obtain materials with excellent antibacterial properties and therefore with high wound healing capacity [16,41,42]. PVA/CS/AgNPs nanocomposite hydrogels were usually obtained by the reduction of silver ions inside the hydrogel [8,43,44], or by the formation of AgNPs simultaneously with the cross-linking induced by gamma or microwave irradiation [16,45]. Nevertheless, these methods do not allow accurate control of the amount of incorporated AgNPs.

Another approach supposes the addition of preformed unstabilized ZnO or Ag/TiO_2_ nanoparticles to the PVA/CS hydrogel [42,46]. However, this procedure needs a very good dispersion of the particles in the precursor’s mixture. Moreover, nanoparticles can easily escape from the polymeric network, and can also determine the reduction of the effective cross-linking. The formation of PVA-stabilized AgNPs by microwave irradiation followed by their mixing with CS solution was performed by Nguyen and collaborators [41,47], but in this case, silver ions were not removed from the PVA–AgNPs solution and the concentration of AgNPs in the obtained hydrogels remained unknown.

Therefore, the objective of the present work was to obtain composite hydrogels with antibacterial properties containing a well-determined amount of AgNPs, uniformly distributed in the polymeric network, using eco-friendly methods both in the preparation of AgNPs and hydrogels. The hypotheses of this work were: (i) the use of OA in the synthesis process of PVA/CS hydrogels to determine an additional physical cross-linking of CS chains that allows to obtain hydrogels with high content of polysaccharide; (ii) the coating of AgNPs with chitosan (used in the synthesis of nanoparticles both as reducing agent and stabilizer) that can be further involved in physical crosslinking with OA and therefore can contribute to the fixation and retention of nanoparticles in the hydrogel.

## 2. Results and Discussion

### 2.1. Synthesis of Chitosan-Capped Silver Nanoparticles (CS-AgNPs)

It is well known that CS can act as a reducing agent for the obtaining of AgNPs, but is also found on the surface of AgNPs, acting as stabilizing agent [19,20,21]. In acidic pH, when the amine groups of CS are in protonated form (–NH_3_^+^), the reduction of Ag^+^ to Ag^0^ by the lone pair electron of oxygen in CS (–OH) requires high temperatures [21]. That is why the solution of CS in acetic acid with added AgNO_3_ was kept at 90 °C for 18 h to obtain CS-AgNPs. After that, in order to separate the CS-AgNPs from the ions (acetate, Ag^+^, NO_3_^–^), NaOH was added. By increasing the pH, not only does the precipitation of CS chains take place, but also a further reduction of Ag^+^ ions by the nitrogen atom from the –NH_2_ groups of CS [48]. The precipitated CS-AgNPs were then separated by centrifugation and purified by dialysis.

**Figure 1:**
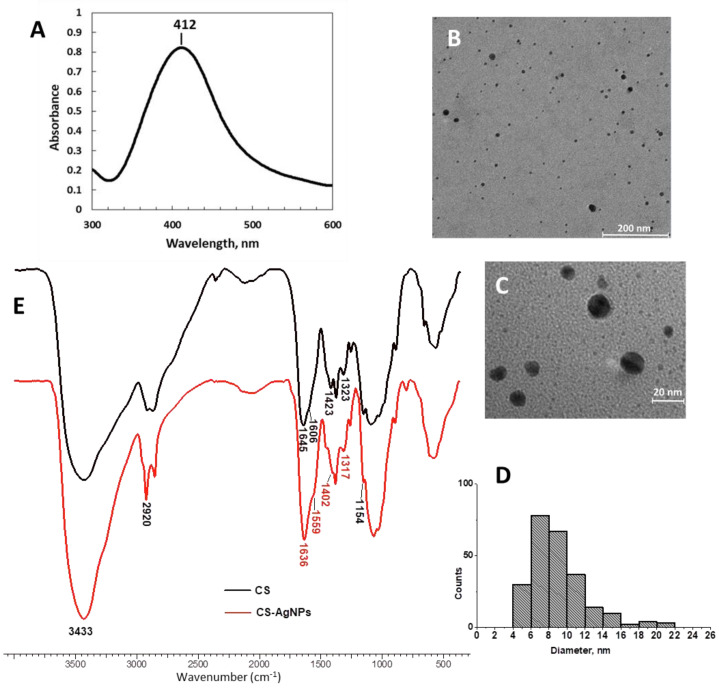
UV–Vis absorption spectra (**A**), TEM images (**B**,**C**), and the size histogram for the CS-AgNPs (**D**). FT-IR spectra of CS (black) and CS-AgNPs (red) (**E**).

In the UV–Vis spectra of CS-AgNPs in aqueous solution (Figure 1A), a single absorption band centered at 412 nm was observed due to the surface plasmon resonance of the nanoparticles, which is characteristic for small spherical silver colloids [49,50]. TEM images (Figure 1B,C) confirmed the spherical shape of the metallic particles. The size of most AgNPs ranged from 4 to 16 nm, but larger particles with diameter up to 22 nm were also present (Figure 1D). The content of Ag in CS-AgNPs, determined by AAS, was 18 wt% (±0.6%), meaning that CS on the surface of nanoparticles represents around 82 wt%.

The FTIR spectra of CS and CS-AgNPs are presented in Figure 1E. The large band from 3000–3600 cm^−1^ in the spectrum of CS, assigned to the stretching vibration of the N–H and O–H bonds, was not as broad in the spectrum of CS-AgNPs, indicating that the amine and hydroxyl groups were affected by the silver attachment. Some bands from the spectrum of CS: 1645 cm^−1^ (C=O stretching, amide I), a shoulder at 1606 cm^−1^ (N–H bending), 1423 cm^−1^ (C–N stretching coupled with N–H plane deformation), and 1323 cm^−1^ (C–N stretching of the amino groups) shifted in the spectrum of CS-AgNPs to 1636, 1606, 1402, and 1317 cm^−1^, respectively_._ These blue-shifts can be due to the interaction between silver and nitrogen atoms from primary amine and amide groups [19,20,51]. Other bands from the CS spectrum at 2920 cm^−1^ (C–H symmetric stretching), 1382 cm^−1^ (CH_3_ symmetric deformation), and 1154 cm^−1^ (C–O–C bridge anti-symmetrical stretching) were not changed, being not sensitive to the metal nanoparticle surface [18,49].

It is known that the dimension of the AgNPs influences the antimicrobial activity: the smaller nanoparticles with higher specific surface area are more effective against bacteria compared to larger nanoparticles [52]. The proposed mechanisms for the antimicrobial activity of AgNPs are (i) the action of the Ag^+^ ion released by the nanoparticles that interact with the sulfur proteins from the cytoplasm and cell walls and can also form complexes with nucleic acids; (ii) the generation of free radicals and reactive oxygen species which induce damage to the bacterial membrane and alteration of DNA; (iii) the interaction of AgNPs with the bacterial membrane and the penetration in the cell [2]. CS is known to have antibacterial activity due to its polycationic structure: the protonated amino groups of CS interact with the negative charge molecules on the surface of bacterial cells [53]. Combining different mechanisms of antibacterial action, CS-AgNPs are known to have a higher antibacterial efficacy compared to simple CS [18,20].

The antimicrobial efficacy of the synthetized CS-AgNPs with 18 wt% Ag was quantified by determining the minimum inhibitory concentration (MIC). MIC values were found to be 5.47 μg/mL for *S. aureus* and 2.73 μg/mL for *K. pneumoniae* (data not shown). The values are similar to those reported in the literature [18,20].

### 2.2. Preparation of PVA/CS/OA Hydrogels

Physically cross-linked hydrogels obtained by freeze–thawing (F-T) technique display macroporous structures with highly interconnected pores. This eco-friendly method consisted of freezing the CS/PVA mixture solution, when the formation of ice crystals forced the polymers to concentrate in an un-frozen water phase. In this polymer-rich phase, physical interactions (hydrogen bonds, PVA crystallization) take place between polymeric chains. The melting of the ice crystals determines the formation of a polymer-poor phase in the meshes of the network. Successive F-T cycles result in ice crystals formation and thawing mainly in the network pores, in the disruption of the hydrogen bonds between polymers and water, and in the formation of further physical intermolecular interactions that reinforce the network structure [33,54]. The presence of CS in PVA cryogels was proved to reduce the crystallinity degree of PVA [32] and to decrease the cross-linking density of the hydrogels [31,33]; however, hydrogels with high CS:PVA ratio cannot even be obtained [31,33].

By replacing the acetic acid with a dicarboxylic acid for the solubilization of CS, the cross-linking of the polysaccharide through ionic cross-linking and hydrogen bonding occurs, with the formation of CS hydrogels by freeze-gelation method in the absence of PVA [38,39]. Among the dicarboxylic acids, OA has the smallest size; therefore, the high influence between the adjacent carboxylic groups determines high dissociation constants of OA (pKa_1_~1.2 and pKa_2_~4.2). Circular dichroism studies on CS solubilized in several dicarboxylic acids showed that OA can strongly solvate CS chains and can overcome the hydrophobic chain associations, resulting in the destruction of the ordered helical structure of CS in solution [55]. Hamdine et al. [56,57] demonstrated that the physical gelation of CS in OA is not due to the ionic bridging, but to ionic interactions between the first dissociated carboxylic group of OA and amino group from CS and to the hydrogen bonds between the second non-dissociated –COOH group of OA and the non-reactive parts of CS (such as C(6)OH, C(3)OH and glycosidic oxygen sites).

Consequently, for the obtaining of PVA/CS/OA hydrogels by F-T method, CS was first solubilized in OA, resulting a 3% CS solution, then mixed with 3% PVA solution. The pH of the final solutions obtained at different OA:CS ratio (0.5–1.25:1, wt:wt) (Table 1) was between 1.8 and 1, indicating that the second carboxylic group of the acid was not dissociated.

**Figure 2:**
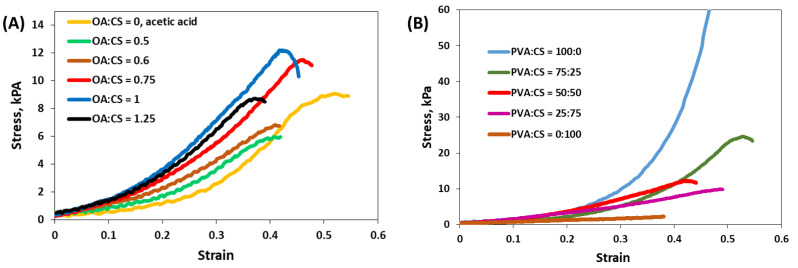
Stress–strain compressive curves for the PVA/CS/OA hydrogels: (**A**) the influence of the OA:CS wt. ratio for hydrogels obtained with PVA:CS = 50:50 (wt:wt) after 7 F-T cycles; (**B**) the influence of PVA:CS ratio for hydrogels obtained with OA:CS = 1:1 (wt:wt) after 7 F-T cycles.

Compression modulus values of the hydrogels obtained right after freeze–thawing cycles can be used as an indicator of physical cross-linking resulting from freeze–thaw cycles [58] (in the absence of the interactions determined by the drying of the samples at room temperature or by lyophilization [59]). Significant compression curves of the PVA/CS/OA hydrogels are shown in Figure 2, and the calculated compression moduli are presented in Table 1 together with the ultimate compressive strength. The influence of the number of F-T cycles, OA:CS ratio, and PVA:CS ratio on the compression and gel fraction was pursued.

The gel fraction (GF) can be also used as a measure of the cross-linking density for the PVA/polysaccharide hydrogels obtained by F-T method [31,33].

With the increase of the number of F-T cycles from three to five, the compression modulus, the ultimate strength, and the GF increase abruptly; while a further increase of the number of F-T cycles causes a reduced augmentation of these parameters. Therefore, most of the physical interactions are formed in the first five cycles (Table 1); however, the hydrogels prepared by a number of F-T cycles smaller than three are too soft and cannot be manipulated.

The ratio between OA and CS also influences the mechanical properties of the hydrogels (Figure 2A); they become stiffer with the increase of the OA amount until 1:1 weight ratio, and the Young modulus and compressive strength increase (Table 1). A further increase of OA amount at OA:CS = 1.25:1 determines a slight decrease of the cross-linking degree. However, it must be taken into account that the OA:CS weight ratios of 0.5:1 and 1:1 correspond to a molar ratio between carboxylic groups of the cross-linker and the CS’s amino groups [HOOC-COOH]:[NH_2_] of 1.15:1 and 2.3:1, respectively. If the cross-linking consisted of ionic bridging, the maximum of the cross-linking would appear at [HOOC-COOH]:[NH_2_] = 0.5:1. Nevertheless, CS is not soluble at this OA content because only one of the carboxylic groups from the OA is dissociated and assures the solubilization of CS. The influence of the OA was not so visible on the GF (Table 1), probably due to the freeze-drying process that facilitated further interactions between the polymeric chains, or because the excess of OA was eliminated by the washing procedure. Hydrogels were also obtained from the same polymers (CS and PVA) and by the same procedure, with CS solubilized in acetic acid; however, in this case, the GF and the elastic modulus were very low.

The ratio between the PVA and CS deeply influenced the shape of the compression curves, as seen in Figure 2B. The hydrogel obtained only from PVA after 7 F-T cycles has a typical “J-shaped” stress–strain curve, as already reported in the literature [59] and it does not reach the breaking point at 60% compression. With the increase of the CS:PVA ratio, the hydrogels become stiffer, the compression modulus increases, but the ultimate compressive strength decreases. These results are in accordance with other studies which proved that mechanical properties of the freeze–thawed PVA/CS hydrogels decreased with the increase of the CS amount [31,33,35].

As it is also shown in Table 1**,** the GF decreased with the decrease of the PVA:CS weight ratio and reached a value of 56% for PVA:CS = 1:1. This gel fraction is relatively low, but acceptable for this concentration of polymers (3%; 1.5% CS and 1.5% PVA in the initial mixture) and in the absence of chemical cross-linking. Hydrogels were also obtained only from CS and OA. In this case, the physical interactions between CS chains (hydrogen bonds) and between CS and OA (electrostatic and hydrogen bonds) are strong enough to assure the formation and stability of the network. When acetic acid was used instead of OA to solubilize the CS, the hydrogels formed by F-T the CS acetate solution were too soft and dissolved once they came in contact with water (data not shown). In contrast, when CS was solubilized in OA, the resulted hydrogels (both in the presence and in the absence of PVA) were stable, showing the importance of OA in the cross-linking of CS.

**Figure 3:**
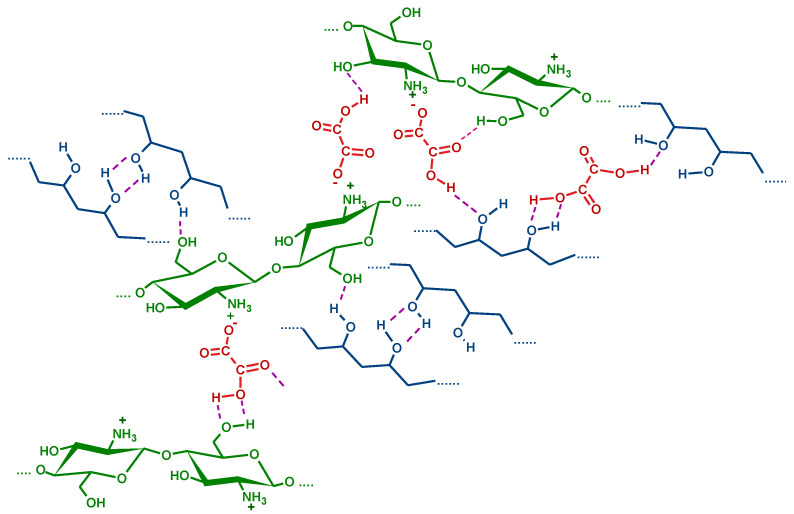
Schematic representation of possible physical interactions in the PVA/CS/OA hydrogels.

The possible interactions between PVA, CS, and OA are hydrogen bonds between PVA chains, ionic interactions between CS and OA, and hydrogen bonds between OA and CS or OA and PVA (Figure 3).

**Figure 4:**
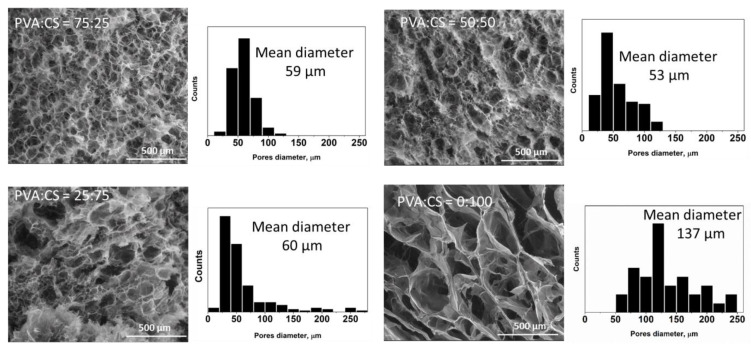
SEM images and the corresponding pore size distribution diagrams for the lyophilized hydrogels with different PVA:CS (wt:wt) ratios obtained with CS:OA = 1:1 (wt:wt) and 7 F-T cycles.

The morphology of PVA/CS/OA hydrogels with different weight ratios between PVA and CS was examined by scanning electron microscopy (SEM) (cross-section of the hydrogels previously washed and dried by lyophilization). The obtained images and the distribution of the pores diameters are presented in Figure 4. The porous structure and the interconnected pores, characteristic for F-T technique, offer a high permeability for gases and water vapor required for wound-dressing applications. The hydrogel with PVA:CS ratio of 75:25 has a regular pore distribution with a mean pore diameter of 59 μm. With the increase of the CS content, the pore distribution became wider because CS reduced the entanglement of the PVA chains [60] and the formation of the PVA crystallites [32]. The increase of the porosity of freeze–thawed PVA/CS hydrogels with the increase of the CS content was also observed in the literature [31,32,34]. The hydrogel obtained only from CS and OA has a sponge-like structure with higher pores diameter (137 μm) and thicker walls compared to PVA/CS/OA hydrogels.

In order to verify the composition of the hydrogels after washing (leakage of the non-cross-linked species), the content of CS in purified samples was determined by ninhydrin assay and the results are presented in Table 2. With the decrease of the PVA:CS ratio in the precursors mixture, the CS content deviated from the theoretical values, and this increase was due to the leakage of OA and PVA during the purification. The leakage of the excess of OA was also observed for the hydrogel obtained only from CS and dicarboxylic acid.

Hydrogels obtained by using a PVA:CS weight ratio of 50:50, OA:CS weight ratio of 1:1, and 7 F-T cycles were chosen for further experiments due to the good balance between the mechanical properties and the CS content.

### 2.3. Preparation of Composite Hydrogels Containing AgNPs

**Figure 5:**
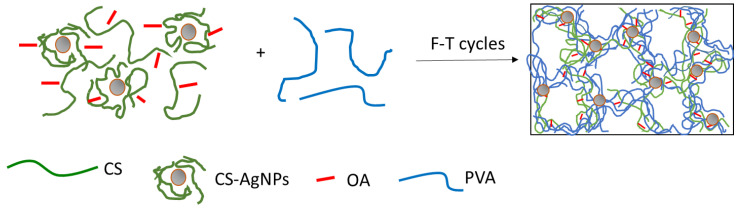
Schematic representation of the preparation of composite hydrogels with embedded AgNPs.

The incorporation of CS-AgNPs into the PVA/CS/OA hydrogels was performed by an ex situ method in which CS-AgNPs were dispersed in the CS solution before mixing with the PVA solution. The concentration of total CS was maintained at 1.5% in the mixture (taking also into account the CS from CS-AgNPs). The ratio between PVA and CS was maintained at 50:50 and the ratio between the total CS and OA at 1:1. The amount of CS-AgNPs increases from sample H-Ag1 to sample H-Ag5, as shown in Table 3. The CS from the surface of AgNPs is involved in the physical interactions with OA and PVA, so the AgNPs are entrapped in the hydrogel, not only in the pores, but also in the walls of the polymeric network (Figure 5).

**Figure 6:**
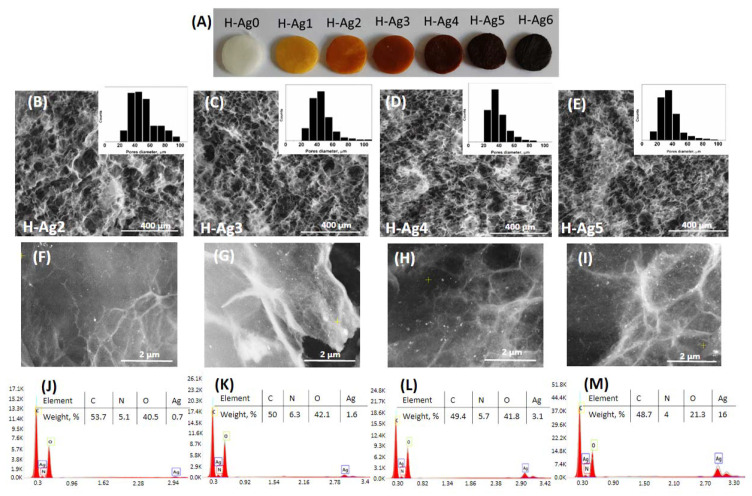
Optical images of the hydrogels with increased silver content (**A**). SEM images (cross-section) at 250× magnification together with the corresponding pore distribution diagrams (**B**–**E**). SEM images at 10,000× magnification, and the energy-dispersive X-ray spectroscopy (EDX) spectra of the lyophilized hydrogels with different contents of AgNPs: H-Ag2 (**B**,**F**,**J**), H-Ag3 (**C**,**G**,**K**), H-Ag4 (**D**,**H**,**L**), and H-Ag5 (**E**,**I**,**M**).

This method allowed the incorporation of the desired amount of AgNPs in the hydrogels. The results from AAS (Table 3) show that the purified hydrogels contain slightly higher amounts of silver than the theoretical values, and this increase can be due to the leakage of OA and PVA from the hydrogels. Moreover, the CS-AgNPs were not removed by the washing procedure, and with the increase of the silver content, the color of the hydrogels changed from yellow to dark brown (Figure 6A).

The elastic modulus and the compressive strength (presented in Table 3) were not influenced by the incorporation of small amounts of AgNPs (H-Ag1 to H-Ag4); but, with further replacement of CS with CS-AgNPs, the mechanical properties decreased. The same trend was observed for the gel fraction. This behavior can be attributed to the fact that CS interacting with AgNPs has a more collapsed/compact chain conformation compared to free CS. In addition, some of the amino groups of CS are involved in the interaction with AgNPs, having fewer groups available for the network formation. This is the reason for the slight decrease of the GF when the amount of CS-AgNPs increased in the detriment of free CS.

The swelling ratio of the hydrogels decreases with the increase of AgNPs amount, especially at high silver contents (Table 3), and this can be explained by the hydrophobicity of silver nanoparticles [61]. The swelling ratio is higher at pH = 5.4 compared to pH = 7.4 due to the protonation of the amino groups from CS in acidic conditions which leads to an increased swelling.

The SEM images of the hydrogels (cross-section) with different amounts of AgNPs are presented in Figure 6. The average pore diameter decreases slightly from 51 μm in the case of H-Ag2 hydrogel to 36 μm for the H-Ag5 sample. It should be noted that the hydrogel samples were first washed with water for three days and then dried by lyophilization, so the decreased porosity is also due to the decreased swelling ratio of the composite hydrogels (see Table 3). This result is in contradiction with some works from the literature where the addition of metallic nanoparticles in freeze–thawed PVA/CS hydrogels determines the increase of the porosity [42].

The small AgNPs can be observed in the SEM images at high magnification (Figure 6F–I), and their density increased from H-Ag2 to H-Ag5. The nanoparticles distribution in hydrogels is uniform without any agglomeration. The elemental composition of the nanocomposite hydrogels was also investigated using EDAX (Figure 6J–M). The determined Ag contents were in accordance with the values obtained from AAS (Table 3) with the exception of H-Ag5 sample, where EDAX showed a higher amount of silver. It should be taken into consideration that the composition determined by EDAX is only on the surface of the network, and the hydrogen content is not taken into account by this method.

**Figure 7:**
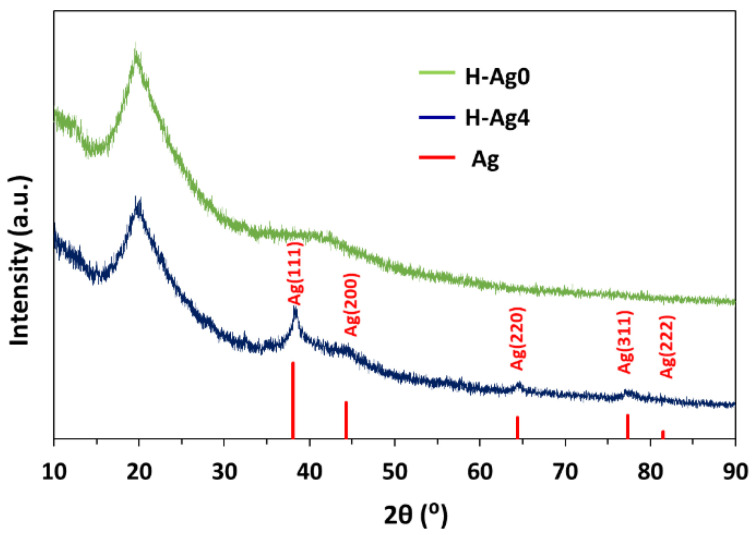
XRD patterns of the PVA/CS/OA hydrogels with (H-Ag4) and without AgNPs (H-Ag0), together with the reference patterns of silver (space group 225:Fm-3m, Card No. 9008459).

The crystalline structure of the composite hydrogels with embedded AgNPs was studied by XRD (Figure 7). The XRD spectrum of H-Ag0 hydrogel with 50:50 wt ratio between PVA and CS presents a major peak at 2θ = 20.2° due to the crystalline structure of both CS and PVA [62,63] and a broad peak at 2θ which ranges from 35° to 50° due to the amorphous region of CS and also to the semicrystalline nature of PVA in the hydrogels [62]. In the XRD spectrum of the hydrogel with embedded AgNPs (H-Ag4 sample), the diffraction peaks characteristic for pure metallic silver are observed. The peaks at 38.3°, 44.3°, 64.4°, 77.3°, and 81.5° correspond to the (111), (200), (220), (311), and (222) reflection planes of crystalline face-centered cubic Ag crystals [12,43].

**Figure 8:**
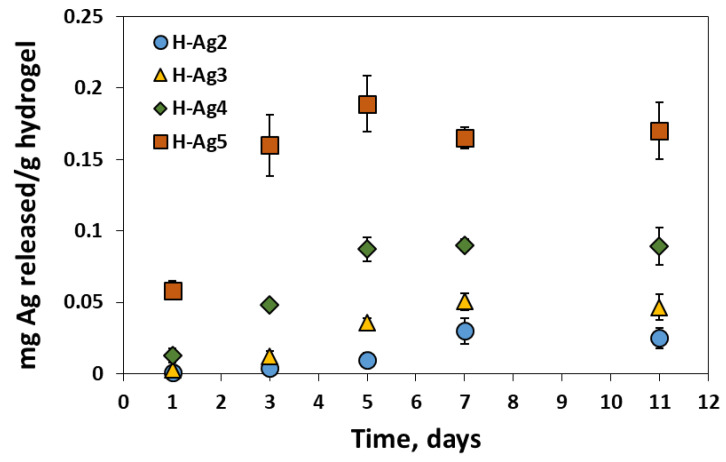
Release profiles of silver from the composite hydrogels in simulated physiological conditions (PB pH = 7.4 and 37 °C).

The release of silver from the composite hydrogels was studied in simulated physiological conditions (PB pH = 7.4, 37 °C) and the results are shown in Figure 8. As expected, the hydrogels with a large amount of embedded CS-AgNPs release high amounts of silver in the buffered solution. The diffusion of silver continues almost linearly for the first 7 h, after which it reaches a plateau. After 11 days, the hydrogels do not release more than 0.4% from the incorporated silver, showing that AgNPs are well trapped in the polymeric matrix.

### 2.4. Antibacterial Studies

**Figure 9:**
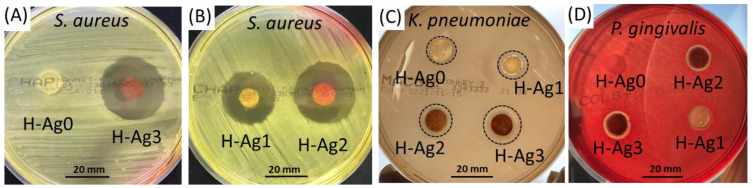
Antibacterial activity of composite hydrogels against *S. aureus* (**A**,**B**), *K. pneumoniae* (**C**), and *P. gingivalis* bacteria (**D**).

The antibacterial activity of nanocomposite hydrogels is an important factor for determining their application for controlling wound infection or for the treatment of periodontitis. Agar disc-diffusion method was used to evaluate the antimicrobial activity of hydrogels against *Staphylococcus aureus* (Gram-positive bacteria), *Klebsiella pneumoniae*, and *Porphyromonas gingivalis* (Gram-negative bacteria) (Figure 9). By increasing the content of embedded AgNPs in hydrogels, the inhibition zone increases (Table 4). The antibacterial effectiveness of the nanocomposite hydrogels was the highest for *S. aureus*. In this case, the sample without silver (H-Ag0) does not have a detectable antimicrobial activity, but the sample with low content of silver (H-Ag1 containing 0.33% Ag) has a high inhibition zone diameter (22 mm) (Figure 9B). A similar antibacterial activity against *S. aureus* was observed for PVA/CS hydrogels loaded with AgNPs that were obtained in the same time with the cross-linking reaction initiated by microwave irradiation [16,41].

In the case of *K. pneumoniae*, the inhibition zone diameter increases from 13 mm for H-Ag0 to 17.5 mm for H-Ag3 hydrogel. The hydrogels with low silver content (H-Ag2 and H-Ag3) release very low amounts of silver in the first day (Figure 8), but these seem to be enough to kill the bacteria in contact with and in the proximity of the hydrogels.

*P. gingivalis* is the major anaerobic oral pathogen which contributes to chronic periodontitis, and AgNPs are known to have antimicrobial activity against these bacteria [64,65]. Therefore, H-Ag1–H-Ag3 samples are effective against *P. gingivalis*, even if the inhibition diameters are small (13 mm for H-Ag3) (Figure 9D). Based on these results, the composite hydrogels can be proposed for the treatment of periodontitis.

### 2.5. In Vitro Cytotoxicity Evaluation

**Figure 10:**
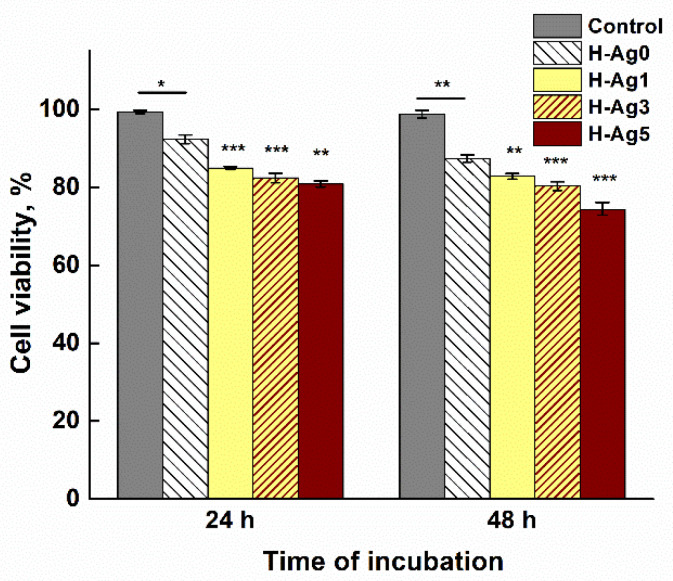
Viability of human dermal fibroblasts cells incubated in the absence (control) and in the presence of hydrogels with different content of silver (*p < 0.05, **p < 0.01, ***p < 0.001).

In addition to antibacterial efficacy, a hydrogel used for wound or periodontitis treatment should have a good biocompatibility. It is known that AgNPs are toxic by themselves and also due to the toxicity of the silver ions released from these nanoparticles [66]. The incorporation of AgNPs into a polymeric network can prevent the release of the nanoparticles and can slow down the release of Ag^+^ cations produced by oxidation of AgNPs inside the hydrogels. For the assessment of the in vitro cytotoxicity of the PVA/CS/OA hydrogels with and without embedded AgNPs, human dermal fibroblast cells were used, and the viability of the fibroblasts after 24 and 48 h of incubation was evaluated with the MTT colorimetric assay. The results are presented in Figure 10.

The cell viability in contact with H-Ag0 hydrogel was around 92% after 24 h and 87.5% after 48 h. In contact with the hydrogel with the lowest content of AgNPs, H-Ag1, the viability of the cells decreased to 85% after 24 h and around 83% after 48 h. As expected, with further increase of the AgNPs amount, the cell viability decreased. The hydrogels have a low toxicity, the cell viability in the presence of AgNPs-containing hydrogels being higher than 80%, with the exception of H-Ag5 sample at 48 h incubation. This low cytotoxicity can be also explained by the very low percent of silver released from the hydrogels after 28 or 48 h.

Compared to our previous work, where AgNPs were immobilized into PVA/CS hydrogels by the immersion of the preformed hydrogels into AgNO_3_ solution and the reduction of silver ions [12], these hydrogels with embedded AgNPs have a low antimicrobial effect but also a low cytotoxicity. This behavior can be explained by the fact that silver ions were released more slowly from the polymeric network where the AgNPs coated with CS were entrapped in the walls of the hydrogel.

## 3. Conclusions

It was demonstrated that OA has an important role in the formation of PVA/CS hydrogels because of its interactions through ionic linkages with CS chains and hydrogen bonds with CS and PVA chains. The number of F-T cycles and the ratio between PVA:CS also influenced the physical cross-linking of the PVA/CS/OA hydrogels, as demonstrated by uniaxial compression tests and gel fraction measurements. The addition of OA allows the formation of hydrogels with high CS:PVA ratio.

Small spherical AgNPs with an average diameter of 9 nm were obtained in diluted aqueous CS solution using the cationic polysaccharide as reducing agent and stabilizer. The preformed organic/inorganic composite nanoparticles containing 18 wt% silver and having antibacterial activity were incorporated in PVA/CS/OA hydrogels. The presence of AgNPs in composite hydrogels was demonstrated by XRD, EDX, and AAS. The addition of small amounts of CS-AgNPs did not influence the physicochemical properties of the hydrogels (mechanic properties, swelling ratio) but assured an antimicrobial activity against *S. aureus*, *K. pneumoniae,* or *P. gingivalis*. The addition of CS-AgNPs in high amounts leads to an increased hydrophobicity of the nanocomposite hydrogels and also to an increased cytotoxicity.

The proposed method allows to obtain hydrogels with the desired content of AgNPs. The CS chains interacting with metallic nanoparticles in CS-AgNPs are also involved in the physical interactions, with OA and PVA assuring the fixation and retention as well as uniform distribution of nanoparticles in the polymeric network. That is why the amount of silver released in simulated physiological conditions (PB with pH = 7.4, 37 °C) is very low (under 0.4% after 11 days) even if the hydrogels are macroporous with an average pores diameter ranging between 30 and 50 μm. The low cytotoxicity and the antibacterial efficacy of the nanocomposite H-Ag2 and H-Ag3 hydrogels with 0.7 and 1.4% Ag recommend their use in wound and periodontitis treatment.

## 4. Materials and Methods

### 4.1. Materials

Chitosan (80% deacetylated, Mv = 240,000 g/mol), PVA (Mowiol^®^ 20–98, Mw~125,000 g/mol, 98–98.8 mol% hydrolysis), and silver nitrate 99% (AgNO_3_) were purchased from Sigma-Aldrich Co. (St. Louis, MO, USA). The molar mass of chitosan, determined by viscometric measurements in 2% CH_3_COOH with 0.2M CH_3_COONa [67], was Mv = 240 kDa, and the deacetylation degree determined by NMR was 80.13%. Oxalic acid anhydrous 98% (OA) was purchased from Thermo Fisher GmbH (Dreieich, Germany).

### 4.2. Methods

#### 4.2.1. Preparation of CS-AgNPs

Chitosan (0.5 g) was dissolved in an aqueous solution of acetic acid 1%, v/v (100 mL) overnight. AgNO_3_ aqueous solution 80 mM (40 mL) was added to the chitosan solution under stirring, and then the mixture was kept for 18 h at 90 °C, in the dark. The formation of AgNPs was indicated by the change of the solution color from yellow to brown. For the precipitation of CS-AgNPs, NaOH 0.5 M was then added until the solution pH was around 9. The precipitate was collected by centrifugation at 4500 rpm for 30 min, washed with 100 mL water, one time, to remove the excess of NaOH, and centrifuged again in order to remove the Ag ions or Ag atoms that are not bound to the CS chains. The CS-AgNPs composite was then purified by dialysis against water by using a dialysis bag (molecular weight cut off 10–12 kDa) and recovered by freeze-drying.

#### 4.2.2. Preparation of PVA/CS/OA Hydrogels

A 3% PVA aqueous solution (w/w) was obtained by dissolving the PVA in water for 1 h at 90 °C. Aqueous solutions of 3% CS (w/w) containing different amounts of oxalic acid (OA:CS = 0.5, 0.75, 1, or 1.25, w/w) were obtained by intense stirring of components for 18 h. Both CS and PVA solutions were cooled down at room temperature, then mixed at different weight ratios, and stirred for 4 h. Finally, solutions were degassed, poured into Petri dishes of 5 cm diameter, and subjected to 7 freeze–thawing cycles (freezing at −20 °C for 18 h and thawing at room temperature for 6 h). Some samples of hydrogels were used as such, without purification for the compression tests. For other samples, hydrogels were lyophilized at −57 °C and 0.045 mbar for 48 h, by using an Alpha 1-2 LD Martin Christ freeze-dryer (Osterode am Harz, Germany), washed with water for 3 days, changing the water every day, and then lyophilized.

#### 4.2.3. Preparation of Composite Hydrogels Containing AgNPs

CS-AgNPs were dispersed into a solution of CS, solubilized with OA. The amount of CS, both in the nanoparticles and in the hydrogel, was calculated for the total concentration of CS to be 3%, and the ratio between Ag and total CS to vary between 0.6% and 18% (w/w). After mixing with 3% PVA solution, the ratio between Ag and polymers (CS and PVA) was 0.3, 0.6, 1.2, 2.4, 4.8, and 9%. The mixed solutions were subjected to 7 F-T cycles and then lyophilized.

#### 4.2.4. Physicochemical Characterization

The UV–Vis spectra of CS-AgNPs in aqueous solution with 0.1% acetic acid were recorded using an Evolution 201 UV–Visible Spectrometer (Thermo Fisher Scientific, Waltham, MA, USA). FT-IR spectroscopy was performed using a VERTEX 70 FT-IR spectrometer (Bruker, Ettlingen, Germany). The morphology of CS-AgNPs was analyzed by TEM using a Hitachi High-Tech HT7700 microscope (Tokyo, Japan) operated in “high contrast” mode and at a 100 kV acceleration potential. Samples were applied from aqueous suspension (1 mg/mL) to 300 mesh copper grids, coated with carbon and dried under vacuum.

The silver content was determined by AAS using a ContrAA 800 spectrometer (Analytik Jena, Jena, Germany) equipped with a xenon short-arc lamp. The solutions were introduced by means of AS-FD autosampler (Analytik Jena, Jena, Germany), and the measurements were carried out in air/acetylene flame at 328 nm. In order to determine the amount of silver in CS-AgNPs, the nanoparticles were dispersed in 5% HNO_3_ aqueous solution, then diluted with water so that the silver concentration would be in the range of the calibration curve (0–2 mg/L). For the determination of Ag in the PVA/CS/OA-AgNPs composite hydrogels, the samples (10 mg) were first digested in 65% HNO_3_ (3 mL) at room temperature for 24 h, then diluted with water prior to elemental analysis.

Compression tests of the hydrogels right after F-T cycles were performed using a texture analyzer (Brookfield Texture PRO CT3^®^, Brookfield Engineering Laboratories Inc., Middleboro, MA, USA). The exact dimensions of the hydrogels samples with cylindrical shape (around 16 mm diameter and 7 mm height) were measured with a digital caliper, then compressed between two parallel plates with a compression rate of 0.2·10^−3^ m/s up to 80% deformation. Prior to each test, the samples were preloaded with a 0.06 N load. The strain is calculated as ratio between the change in height and the original height, and the stress was calculated as ratio between the compression force (N) and the initial cross-sectional area of the sample (m^2^). The elastic modulus, E, was calculated from the slope of the strain–stress curve at the initial linear segment (0–15% strain). The ultimate compressive strength was obtained from the stress–strain curve as the maximum stress before the failure of the hydrogel. Three to five replicates were used and the data were averaged.

Gel fraction. The dry hydrogels obtained after F-T cycles and lyophilization were weighed (*W*_0_), and then washed in distilled water for two days in order to remove the leachable polymeric chains and OA. The hydrogels were then freeze-dried and weighed again (*W_w_*). The gel fraction was calculated as

$$GF=\frac{w_{w}}{w_{0}}\times 100$$

For the determination of the swelling ratio (*SR*), the dried hydrogels samples were soaked in buffer solutions for 24 h when an equilibrium swelling was reached. The samples were withdrawn; the excess of superficial water was removed with a filter paper, and then weighed. The *SR* was calculated as

$$SR=\frac{w_{s}-w_{d}}{w_{d}}\times 100$$

where $w_{d}$ and $w_{s}$ are the weight of the dry and the swollen hydrogel, respectively. Phosphate buffer solutions (PB) with pH = 7.4 and 5.4 (66.7 mM, KH_2_PO_4_ and Na_2_HPO_4_), were used for the swelling of the hydrogels.

The morphology of the freeze-dried hydrogels was evaluated by scanning electron microscopy using a Quanta 200 (FEI Company, Brno, Czech Republic) electron microscope. The hydrogels, washed with water and dried by lyophilization, were cut to expose their inner structure and used for SEM studies in low vacuum, using SE (secondary electrons) mode, and an acceleration voltage of 20 kV. The diameter of the pores was measured using ImageJ 1.52 version software. For elemental composition and for high-magnification images (10,000×), a Verios G4 UC scanning electron microscope (Thermo Fisher Scientific, Brno, Czech Republic) equipped with an Octane Elite SDDs EDX detector (Ametek, Berwyn, PA, USA) was used. The images with high magnification of the hydrogels containing silver nanoparticles were obtained in high vacuum in SE mode at 20 kV acceleration voltage.

Ninhydrin assay was performed in order to determine the content of CS in hydrogels [68,69]. The ninhydrin reagent solution was prepared according to literature [68]. A total of 5 mg hydrogel was swollen in 5 mL 0.5% acetic acid overnight, then 5 mL ninhydrin agent was added and the mixture was heated in boiling water for 30 min, when the dissolution of the polymeric network took place together with the ninhydrin reaction with the primary amino groups from CS. The obtained blue solution was cooled down, diluted with 50% ethanol: water, and the absorbance at 570 nm was measured. The content of CS was determined using a previously obtained calibration curve in the range 0.1–1 mg/mL CS.

A Miniflex 600 diffractometer (Rigaku, Tokyo, Japan) was used for the X-ray diffraction (XRD) analysis of the composite hydrogel containing AgNPs, and the diffraction peaks were identified using the Crystallography Open Database.

In order to study the release of silver from the hydrogels, PVA/CS/OA-AgNPs hydrogels (15 mg) were immersed in 5 mL PB, pH = 7.4. Five scintillation vials with hydrogel and PB were prepared for every hydrogel type and kept at 37 °C. After 1, 3, 5, 7, and 11 days, the solution was extracted, acidified with 5 μL HNO_3_ 65%, and the concentration of Ag in the solution was determined by AAS. The experiments were performed in duplicate. This method allows the estimation of AgNPs and Ag^+^ ions diffused from the hydrogels.

#### 4.2.5. Antimicrobial Activity

The antimicrobial activity of CS-AgNPs has been investigated against *Staphylococcus aureus* ATCC 25923, Gram-positive bacteria, and *Klebsiella pneumoniae* ATCC BAA-1705, Gram-negative bacteria. For the AgNPs in solution, the minimal inhibitory concentration (MIC) was determined by the tube dilution method [70]. Twofold (1:2) dilutions of CS-AgNPs in concentration ranging from 87.5 to 1.36 mg/mL were prepared (1 mL in each tube) and the tubes were seeded with 0.5 mL of tested microorganisms. The initial bacterial concentration was in the range of 10^5^ to 10^6^ CFU/mL. The optical turbidity of the tubes was evaluated after incubation at 37 °C for 48 h, and the MIC is the lowest concentration of the CS-AgNPs which visibly inhibits the bacterial growth.

The antibacterial activity of the composite hydrogels was tested using the disc diffusion method [71] against *Staphylococcus aureus* ATCC 25923, *Klebsiella pneumoniae* ATCC BAA-1705, and *Porphyromonas gingivalis* ATCC 33377 (a Gram-negative oral anaerobe involved in the pathogenesis of periodontitis). Briefly, the hydrogel discs with 10 mm diameter were first hydrated in sterile distilled water, and then placed on agar plates previously seeded with each strain (McConkey agar for *S. aureus*, Mannitol salt agar for *K. pneumoniae*, or Columbia Agar with 5% Sheep Blood for *P. gingivalis*) (Thermo Fisher Scientific, MA, USA). The diameter of the inhibition zone was measured after incubation at 37 °C for 24 h. The experiments were performed in duplicate.

#### 4.2.6. Cytotoxicity Assay

Cytotoxicity studies were performed according to our previous paper [12] by using MTT assay protocol and human dermal fibroblasts adult cells (HDFa) cultured in DMEM (Dulbecco’s Modified Eagle Medium) supplemented with 10% fetal bovine serum, 1% antibiotics, and 1% non-essential amino acids, at 5% CO_2_ and 37 °C. The sterilized hydrogel samples in the form of discs (4 mm diameter, 2 mm length, and around 1 mg weight) were incubated in the presence of cells on flat-bottom 96-well plates for 24 and 48 h. At the end of the incubation interval, 100 μL of culture medium was replaced with the same volume of fresh medium followed by adding 10 μL of MTT dye (from 5 mg/mL stock) to each well and incubating for 4 h at 37 °C with 5% CO_2_ in the dark. Finally, 90 μL of medium was removed and the formazan crystals, developed as a result of cellular reduction of MTT, were dissolved in DMSO (100 μL) solution and incubated for 10 min at 37 °C. Then, the absorbance at 570 nm was measured using a Multiskan FC automatic plate reader (Biotek, Germany). Each hydrogel sample was tested in triplicate. Cell viability was expressed as % of untreated cells (control) considered 100% viable.

#### 4.2.7. Statistical Analysis

Data were presented as mean ± standard deviation. The ANOVA single factor was performed for statistical analysis and the significance level was considered at *p* < 0.05.

## Figures and Tables

**Table 1:** Influence of the number of F-T cycles, OA:CS, and PVA:CS ratios on hydrogel characteristics.

Preparation			Compression after F-T Cycles		Gel Fraction (%)
PVA:CS (wt:wt)	OA:CS (wt:wt)	Number of F-T Cycles	E (kPa)	Ultimate Compressive Strength (kPa)	
50:50	0.75:1	3	5.0 (±0.4)	2.7 (±0.1)	31.8 (±1.2)
		4	8.3 (±0.15)	5.0 (±0.3	39.6 (±2.1)
		5	10.1 (±0.2)	7.3 (±0.1)	48.9 (±0.8)
		6	10.5 (±0.1)	8.1 (±0.2)	52.6 (±0.8)
		7	11.0 (±0.5)	10.9 (±0.6)	56.0 (±0.7)
50:50	0:1 *	7	3.5 (±0.6)	9.4 (±0.4)	39.9 (±1.9)
	0.5:1		4.5 (±0.9)	5.6 (±0.6)	49.3 (±1.6)
	0.6:1		8.1 (±0.7)	6.8 (±0.3)	53.1 (±0.7)
	0.75:1		11.0 (±0.5)	10.9 (±0.9)	57.3 (±1.2)
	1:1		11.6 (±0.9)	11.8 (±1.1)	56.0 (±0.7)
	1.25:1		10.2 (±2.2)	7.8 (±0.8)	55.2 (±0.9)
100:0	1:1	7	10.6 (±0.4)	-	89 (±0.8)
75:25			7.8 (±0.4)	25.7 (±1.1)	66.2 (±1.6)
50:50			11.6 (±0.9)	11.8 (±1.1)	56.0 (±0.7)
25:75			16.3 (±0.6)	9.8 (±0.5)	52.1 (±1.2)
1:100			5.4 (±1.0)	1.2 (±0.1)	49.2 (±2.2)

* CS was solubilized in acetic acid.

**Table 2:** The content of CS in hydrogels with different PVA:CS:OA ratio.

PVA:CS:OA (wt:wt:wt)	CS Content, wt%	
	Theoretical	Ninhydrin Assay
75:25:25	20	21.3 (±0.7)
50:50:50	33.3	46.9 (±1.8)
25:75:75	42.8	67.7 (±2.1)
0:100:100	50	73.1 (±0.2)

**Table 3:** Composition and physicochemical properties of the composite hydrogels containing CS-AgNPs.

Sample	PVA:CS: CS-AgNPs:OA (wt:wt:wt:wt)	Ag Content (wt %)		Compression after F-T Cycles		Gel Fraction (%)	Swelling Ratio (g/g)	
		Theoretical	from AAS	E (kPa)	Ultimate Compressive Stress (kPa)		PB, pH = 7.4	PB, pH = 5.4
H-Ag0	50:50:0:50	0	-	11.6 (±0.9)	11.8 (±1.1)	56.0 (±0.7)	18.0 (±0.5)	22.3 (±1.1)
H-Ag1	50:48.6:1.7:50	0.2	0.33	11.8 (±0.2	11.8 (±0.8)	56.8 (±0.8)	18.6 (±0.5)	22.2 (±1.0)
H-Ag2	50:47.3:3.3:50	0.4	0.76	11.3 (±0.8)	12.6 (±0.7)	55.5 (±1.1)	18.8 (±0.6)	22.9 (±1.2)
H-Ag3	50:44.5:6.7:50	0.8	1.4	11.6 (±0.4)	13.6 (±0.6)	56.9 (±0.9)	16.5 (±0.5)	22.6 (±0.9)
H-Ag4	50:39.1:13.3:50	1.6	2.8	10.6 (±0.5)	15.3 (±0.5)	54.8 (±0.7)	15.5 (±0.3)	21.9 (±1.3)
H-Ag5	50:28.1:26.7:50	3.1	4.4	8.4 (±0.5)	11.5 (±0.2)	47.3 (±1.6)	15.0 (±1.0)	21.6 (±0.9)
H-Ag6	50:9:50:50	8.3	n.d.	6.8 (±0.2)	9.6 (±0.9)	44.7 (±1.3)	14.9 (±0.9)	19.6 (±0.8)

**Table 4:** Diameter of inhibition zone of bacteria in the presence of nanocomposite hydrogels.

	Diameter of the Inhibition Zone (mm)			
Hydrogel	H-Ag0	H-Ag1	H-Ag2	H-Ag3
Organism				
*Staphylococcus aureus*	10	22	26	26
*Klebsiella pneumoniae*	13	14	15	17.5
*Porphyromonas gingivalis*	-	11.5	12	13
